# Simultaneous biofiltration of H_2_S, NH_3_, and toluene using compost made of chicken manure and sugarcane bagasse as packing material

**DOI:** 10.1007/s11356-024-33757-1

**Published:** 2024-06-26

**Authors:** Ana María Guzmán-Beltrán, Diana Vela-Aparicio, Sergio Montero, Iván O. Cabeza, Pedro F. B. Brandão

**Affiliations:** 1 Universidad Nacional de Colombia - Sede Bogotá - Facultad de Ingeniería - Departamento de Ingeniería Química y Ambiental, Av. Carrera 30 #45-03, 111321 Bogotá D.C., Colombia; 2 Universidad Nacional de Colombia - Sede Bogotá - Facultad de Ciencias - Departamento de Química - Grupo de Estudios para la Remediación y Mitigación de Impactos Negativos al Ambiente (GERMINA), Av. Carrera 30 #45-03, 111321 Bogotá D.C., Colombia; 3 Present Address: Universidad Santo Tomás - Facultad de Ingeniería Ambiental - INAM-USTA, Carrera 9#51-11, Bogotá D.C., Colombia; 4 Universidad de la Sabana - Facultad de Ingeniería, Laboratorio de Energía, Materiales y Ambiente, Campus Universitario Puente del Común, Km. 7 Autopista Norte de Bogotá, Chía, Cundinamarca Colombia

**Keywords:** Biofiltration, Compost, Offensive odors, Hydrogen sulfide, Ammonia, Volatile organic compounds

## Abstract

Offensive odors from wastewater treatment plants (WWTP) are caused by volatile inorganic compounds such as hydrogen sulfide and ammonia and volatile organic compounds (VOCs), such as toluene. To treat these pollutants, biofiltration is an effective and economical technology used worldwide due to its low investment and environmental impact. In this work, a laboratory-scale prototype biofilter unit for the simultaneous biofiltration of hydrogen sulfide, ammonia, and toluene was evaluated by simulating the emission concentrations of the El Salitre WWTP Bogotá, Colombia, using a compost of chicken manure and sugarcane bagasse as packing material for the biofilter. The prototype biofilter unit was set to an operation flow rate of 0.089 m^3^/h, an empty bed residence time (EBRT) of 60 s, and a volume of 0.007 m^3^ (6.6 L). The maximum removal efficiency were 96.9 ± 1.2% for H_2_S, at a loading rate of 4.7 g/m^3^ h and a concentration of 79.1 mg/m^3^, 68 ± 2% for NH_3_, at a loading rate of 1.2 g/m3 h and a concentration of 2.0 mg/m^3^, and 71.5 ± 4.0% for toluene, at a loading rate of 1.32 g/m^3 ^h and a concentration of 2.3 mg/m^3^. The removal efficiency of the three compounds decreased when the toluene concentration was increased above 40 mg/m^3^. However, a recovery of the system was observed after reducing the toluene concentration and after 7 days of inactivity, indicating an inhibitory effect of toluene. These results demonstrate the potential use of the prototype biofilter unit for odor treatment in a WWTP.

**Supplementary Information:**

The online version contains supplementary material available at 10.1007/s11356-024-33757-1.

## Introduction

Wastewater treatment plants (WWTP) are sources of offensive odors that cause negative health effects on workers and inhabitants of residential neighborhoods living nearby the plants. These odors are generated by volatile inorganic compounds such as hydrogen sulfide (H_2_S), ammonia (NH_3_), and volatile organic compounds (VOCs) (Iranpour et al. 2005), which need to be treated to comply with air quality regulations. For this purpose, biological technologies such as biofiltration are widely used in Europe and North America because they are easy to implement, have low investment costs, and do not produce pollutant streams compared to physicochemical technologies such as chemical scrubbing and adsorption. Biofiltration is a process based on biological oxidation which takes place in reactor which is packed with media such as compost. As the gaseous emissions pass through the biofilter, they are adsorbed on the biofilm and oxidized by microorganisms (Brancher et al. 2017). In addition, this biotechnology contributes to the 3rd Sustainable Development Goal on good health and well-being, as biofiltration can reduce illnesses from hazardous chemicals and air pollution, such as unpleasant odours, which affect the mental and physical health of people living near the WWTP. It also contributes to the 11th Sustainable Development Goal of making cities and human settlements inclusive, safe, resilient and sustainable because treating these pollutants reduces the environmental impact of cities in terms of air quality.

Considering that the packing material of a biofilter is one of the main factors that affect the degradation of the compounds, a series of characteristics must be met: (1) have a high specific area and porosity for compound adsorption, (2) optimal water retention capacity to avoid water loss, and (3) buffering capacity to avoid abrupt pH changes that could affect microorganisms. Additionally, it may have intrinsic nutrients and microbiota that oxidize the pollutants (Muñoz et al. 2015). For these reasons, compost, soil, peat, and wood chips are commonly used in biofiltration as they fulfil most of the characteristics and are economical and affordable. However, compost has a tendency to compact, break down, or clog, resulting in pressure drop, anaerobic zones, and a reduction in removal efficiency. To avoid these problems, compost is mixed with structuring agents, such as wood chips or bark, perlite, or vermiculite, which improve the maintenance of the bed structure, rigidity, and lifespan (Barbusinski et al. 2017). Compost and lignocellulosic materials have been used to treat a great variety of different pollutants. For example, biofiltration of toluene, cyclohexane, and hexane was studied using packing material made of a mixture of compost and wood chips with an inoculum of activated sludge, achieving a removal efficiency (RE) for toluene of 100% (EBRT: 82 s), for cyclohexane 86 ± 6% (EBRT: 163 s), and for hexane 96 ± 4% (EBRT: 245 s) (Lamprea Pineda et al. 2023). Another example is the treatment of butyric acid and limonene that are produced by organic waste in municipal treatment plants, using sewage sludge compost and wood chips as packing material and EBRT of 60 s. The result was a removal efficiency of between 70 and 100% for butyric acid and less than 70% for limonene (Márquez et al. 2022).

Nevertheless, most of the biofiltration studies related to odor treatment in WWTPs using compost beds have focused on the treatment of H_2_S, as it is the pollutant with the highest concentration in WWTPs (Jiang et al. 2017). As a result, there has been no comprehensive evaluation of the simultaneous biofiltration of of H_2_S with NH_3_ and VOCs, nor of the effects that these may have on the H_2_S removal or interactive effects between pollutants. However, there are several reports on biofiltration of H_2_S and NH_3_. For example, Vela-Aparicio et al. (2022) simulated the concentrations of these compounds emitted at the WWTP-El Salitre of Bogotá and used a compost of manure with sugarcane bagasse as a packing bed. The removal efficiency for both gases was above 98%, but at 75 mg/m^3^ of H_2_S and 5 mg/m^3^ of NH_3_, the removal efficiency decreased in the inoculated biofilter to 90% and 85% respectively, and in the uninoculated biofilter 80% and 75%, respectively. While another study made in China for treatment of H_2_S, NH_3_, and VOCs from a sewage sludge composting facility, required coupling two techniques: biological trickling filtration (with nutrient solution) for hydrophilic compounds and fungal biological filtration for hydrophobic ones, the packing material for both technologies was a 5:4:1 mixture of sewage sludge from a WWTP, its stabilized compost and peanut hull (Tian et al. 2023).

However, few studies have included toluene in the removal by a biofilter since it is the most abundant VOC that is emitted in different municipal WWTPs (Widiana et al. 2019). For example, Galera et al. (2008) is the only report found that used a compost bed (rock wool and compost at a ratio 7:3 by weight) for the simultaneous biofiltration of H_2_S, NH_3_, and toluene. As a result, they obtained a removal efficiency of 100% for concentrations between 50 and 55 ppm (52.0–55.2 mg/m^3^) of each compound. However, these authors reported that at 220 ppm (228.8 mg/m^3^) H_2_S, the toluene removal decreased by more than 50%.

Therefore, the above studies demonstrate the need to evaluate interferences between contaminants, the effects on inlet concentration loading rate, bed moisture, the behavior of degradation products, and the microbial community in laboratory or pilot scale studies. Very few studies have evaluated the simultaneous biofiltration of volatile inorganic and volatile organic compounds that cause offensive odors in a WWTP. For this reason, the novelty of this work is that it assesses a prototype for the biofiltration of H_2_S, NH_3_, and toluene simulating WWTP-El Salitre of Bogotá concentrations (concentrations trends are shown in *Online Resource Fig. S**1*) and transient conditions of moisture loss and high concentration of pollutants. In addition, oxidation products, pH and microbial population fluctuations were analyzed to understand the mechanisms and interactions between the pollutants. In consequence, the results of this study will approximate the real behaviour of a biofilter under WWTP pollutant conditions for possible scaling-up and field application. Additionally, chicken manure compost and sugarcane bagasse (structuring agent) were used as packing materials. This also represents a waste management strategy for these agro-industrial residues and contributes to the circular economy, especially in Colombia where these residues are widely produced and have no second use.

## Materials and methods

### Composting

Composting methodology was performed according to Vela-Aparicio et al. (2020) with some modifications. Chicken manure was collected from a farm located in Chocontá, Colombia, and sugar cane bagasse from Valle del Cauca, Colombia. Material particles above 2 mm particle size were selected by sieving through a 2-mm mesh to avoid compaction during composting and biofiltration. A mix of 6:4 sugar cane bagasse:manure in volume was composted in a 100 L high-density polyethylene reactor (Earthgreen Colombia 2023), which had holes and a vent cone for air circulation (*Online Resource Fig. S**2*). In this work, sugar cane bagasse was used as structural material as reported by Vela-Aparicio et al. (2020). Besides, due to a possible high emission of ammonia (Vela-Aparicio et al. 2020; Bernal et al. 2009), in this study the ratio of manure was set at 40%. The compost was mixed weekly and moisture was adjusted at 40%, monitoring three times a week. The composting process was run for 11 weeks.

### Biofiltration system

**Fig. 1:**
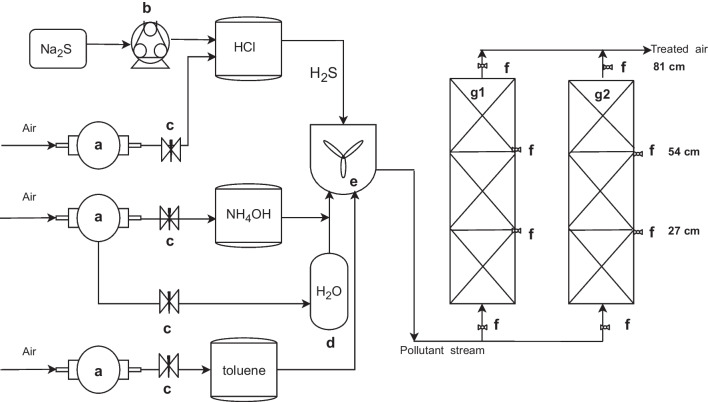
Gas generation and laboratory-scale biofiltration system. a. Vacuum pump; b. Peristaltic pump; c. Valve; d. Humidifier; e. Mixing chamber; f. Sampling port; g1. and g2. Biofilters 1 and 2

The laboratory set-up (Fig. 1) consisted of two biofilters and a gas generation system (*Online Resource Fig. S**3*). The biofilters were built with PVC pipes of 10.16 cm in diameter and 81 cm in height, and were packed with compost to obtain a total volume of 0.007 m^3^ (6.6 L). The biofilters were composed of three sections, each with 27 cm in height that contained a sampling port to measure gas concentrations (*Online Resource Fig. S**4*).

The gas generation system consisted of vessels with H_2_S, NH_3_, and the VOC solutions, connected individually to a vacuum pump (Rocker 300 brand) for air sparging that volatilized the compounds. The VOC chosen for the assessment was toluene because it was identified as one of the most abundant compounds of the pre-treatment zone at El Salitre WWTP by gas chromatography coupled with mass detector analysis (*Online Resource Table* *1*). H_2_S was produced by adding Na_2_S solutions, with a concentration range from 6 to 20 mM, to a 1 M HCl solution using a peristaltic pump (MasterFlex model 7519-15) at 0.024 mL/min flow rate. Then, H_2_S was volatilized by connecting an inverse vacuum pump (Rocker 300 brand) to the vessel. A calibration curve was made between the Na_2_S concentration that was added to the HCl and the H_2_S gas concentration obtained (*Online Resource Fig. S**5*). NH_3_ was volatilized the same way as H_2_S, but from an NH_4_OH 1% v/v aqueous solution. Toluene was volatilized from a 0.5% solution in mineral oil. NH_3_ and toluene concentrations were controlled by a valve placed between the vacuum pump and each container. Additionally, compressed air was injected into a vessel containing water (humidifier) to produce a stream of humid air. The gases and humidifier streams were pumped into a mixing chamber and then to the biofilter. The inlet flow rate was adjusted to 6.5 L/min which was verified with flowmeters (range 0–12 L/min, precision of ± 4%; Flowtron), to obtain a retention time of 60 s. Furthermore, outlet flow was also measured to determine a possible pressure drop.

The biofilters were exposed to the experimental concentrations of H_2_S, NH_3_, and toluene simultaneously (Table 1), which were simulated according to the gas monitoring carried out previously in the pre-treatment area of El Salitre WWTP. In general, H_2_S is the compound that is emitted in the highest concentration and with the least fluctuation compared to NH_3_ and VOCs. Then, for each concentration of H_2_S, the concentrations of NH_3 _and VOCs found in the measurement were related. Subsequently, a graph was made relating NH_3_ and VOCs concentrations to the concentration of H_2_S (*Online Resource Fig. S**1*). Finally, a concentration range was established for each type of emission (Table 1).

**Table 1:** H_2_S, NH_3_ and VOC concentrations used to evaluate biofiltration with compost as packing material at different stages and according to gases measured at the WWTP-El Salitre, Bogotá, Colombia

Phase	Day	H_2_S (mg/m^3^)	NH_3_ (mg/m^3^)	Toluene (mg/m^3^)	Moisture (%)
I	0–25	4.2 ± 0.2	0.9 ± 0.1	5.8 ± 0.8	45
II	26–43	15.3 ± 0.8	2.0 ± 0.1	10.9 ± 2.2	45
III	44–60	34.7 ± 0.9	2.4 ± 0.1	14.8 ± 1.4	45
IV	60–72	48.2 ± 0.5	2.4 ± 0.1	16.4 ± 2.7	50
V	73–83	79.1 ± 3.5	2.4 ± 0.1	22.3 ± 1.9	50
VI	84–89	84.1 ± 3.4	2.4 ± 0.1	40.9 ± 10.0	50
VII	90–103	42.9 ± 1.7	2.4 ± 0.1	15.1 ± 2.8	50

The biofiltration assessment was divided into seven phases (I to VII) according to the increase of H_2_S and toluene concentration, while NH_3_ remained constant from phase II. Specifically, phases V and VI correspond to transient conditions of high loading rates and shock concentrations that are abrupt increases of concentration, usually less frequent during a WWTP operation. Finally, the biofiltration was stopped for 7 days and a system recovery stage (VII) was evaluated, in which the concentrations of H_2_S and toluene were decreased to similar concentrations of phases III and IV (Table 1).

During phases I, II, and III, the initial moisture (dry basis) of the bed was adjusted to 45 ± 3%, while in stages IV, V, VI, and VII, the humidity was adjusted to 50 ± 3% since dry and compact zones were evident in the bed. Furthermore, moisture was monitored every 3 days by calculating the weight difference of each section, and then each section was manually sprayed to adjust the moisture if it was necessary when there was a weight difference and the moisture was below 50%.

### Gas analysis

A portable multi-gas monitor (MultiRAE PGM-6228 RAE Systems) was used to measure H_2_S, NH_3_, and toluene. This monitor detects VOCs total concentration and as toluene was the only VOC of study, it was assumed that the VOC measurement of the monitor was the same concentration of toluene. The concentration range for each compound was H_2_S: 0.1–100 ppm, NH_3_: 1–100 ppm, VOCs: 0.1–5000 ppm, and resolution H_2_S: 0.1, NH_3_: 1, VOCs: 0.1 (Honeywell 2015). Gas sampling was performed at the inlet and the three sampling ports of the biofilter (Fig. 1(f)) by placing a hose between the port and the detector. The ports were covered after the measurement to prevent gas leak. Finally, the measurements were taken once the gas concentration was steady and three times per day.

The mass of contaminants that enter the biofilter per unit of time and volume was calculated using Eq. 1:

1 $$\mathrm{LR}=\frac{C_i\ast {Q}_{\mathrm{gas}}}{{\mathrm{Volume}}_{\mathrm{bed}}}$$

where, *C*_*i*_: gas input concentration (mg/m^3^); *Q*_gas_: flow rate of polluted air (m^3^/h); concentration (mg/m^3^); *C*_*o*_: gas outlet concentration (g/ m^3^); and Volume_bed_: Volume occupied by the bed (m^3^).

Daily, the removal efficiency percentage (*%*RE) was calculated for each gas and section using Eq. 2:

2 $$\%\mathrm{RE}=\frac{\left({C}_i-{C}_o\right)\ast 100}{C_i}$$

where, *C*_*i*_: gas input concentration (mg/m^3^); and *C*_*o*_: gas concentration in each section (mg/m^3^).

Additionally, the elimination capacity (EC) was calculated for each gas only at the outlet or upper biofilter section, using Eq. 3:

3 $$\mathrm{EC}\left(\frac{g}{m^3h}\right)=\frac{\left({C}_i-{C}_o\right)\ast {Q}_{\mathrm{gas}}}{{\mathrm{Volume}}_{\mathrm{bed}}\ast 1000}$$

### Physicochemical analyses of compost

Composting evolution was followed by the biodegradability coefficient (*Km*) that indicates the degradation achieved in composting and its stability in presence of a microbial population. *Km* is defined as the volatile solids lost in the composting process in function to the initial volatile solid of the material mix. This definition is based on the conservation of ash principle and *Km* can be calculated according to Eq. 4 (Haug 1993):

4 $$Km=\frac{\left({OM}_1-{OM}_2\right)\ast 100}{OM_1\ast \left(100-{OM}_2\right)}$$

where,

*OM*_*1*_: initial percentage of organic matter (%); and *OM*_*2*_: total percentage of organic matter (%).

Organic matter (OM) content was determined by drying the compost at 105 ± 5 °C in an oven, which was then calcined at 550 ± 10 °C in a muffle (APHA 2005).

In addition, other characteristics were determined for the final compost product used as biofilter packing material. Moisture was determined in a thermobalance (PCE-MB 120C). Nitrogen, bulk density, pH, conductivity, and density were determined according to the method NTC 5167 (ICONTEC 2011). Nitrogen concentration was characterized by the Kjeldahl method. Bulk density was determined as the ratio of compost mass in 500 mL. pH and conductivity were measured in the 1:10 compost:water extract by a pH meter (Hanna Instruments HI 11310) and electrical conductivity meter (Hanna Instruments HI 99300), respectively. Particle size distribution was determined by the EN 13040 method (CEN 2007). Water holding capacity was determined by soaking compost in water for 24 h, followed by draining and determining the gravimetric difference (Dorado et al. 2010). Buffer capacity or the amount of acid required to reduce a unit of pH was determined by adding different concentrations of H_2_SO_4_ to the compost in a ratio 1:10 (compost:water; w/w), and after 72 h, pH was measured (Costello and Sullivan 2014). In addition, the porosity was calculated according to the “Analytical Methods of Soil Laboratory” of the Agustín Codazzi Geographic Institute (IGAC 2006), by determining the apparent density (Da) and real density (Dr).

Finally, the abundance of bacteria was evaluated by counting colony-forming units per gram of compost (CFU/g) on agar plate. First, an extraction was carried out in 0.85% NaCl saline solutions and then serial dilutions were seeded. For heterotrophic bacteria, TSA medium (Trypticase Soy Agar, Difco) was used and incubated for 48 h at 30 °C (Topp et al. 1998). On the other hand, in the case of sulfur-oxidizing bacteria (SOB), a medium of Na_2_S_2_O_3_ and minerals was used as reported by Cha et al. (1999) and incubated for 10 days at 30 °C. Ammonium-oxidizing bacteria (AOB) were cultured in a media which had (NH_4_)_2_SO_4_, minerals, and a trace element solution of CuSO_4_.5H_2_O 1.57 g/L, (NH_4_)_8_Mo_7_O_24_.4H_2_O 1.1 g/L, CoCl_2_.6H_2_O 1.6 g/L, EDTA 5 g/L, MnCl_2_ 5 g/L, FeCl_3_ 5 g/L (Kim and Ivanov 2000). Likewise, nitrite-oxidizing bacteria (NOB) were cultured in the media which had NaNO_2_, minerals, and the same trace element solution reported by Kim and Ivanov (2000). Both AOB and NOB were also incubated for 10 days at 30 °C.

### Monitoring of oxidation products, toluene, and pH

At the end of phases I, III, VI, and VII, the degradation metabolites of H_2_S (sulfate ion: SO_4_^2−^) and NH_3_ (nitrite ion: NO_2_^−^, and nitrate ion: NO_3_^−^) were determined. These phases were selected for the monitoring of oxidation products because they represent a greater increase in the concentration of H_2_S and NH_3_ gases. For the ion analysis, an aqueous extract was prepared with deionized water from the bed of a 1:10 and then 1:25 dilution and, later, it was analyzed by the method 4110 B of ion chromatography with chemical suppression of the conductivity of the eluent (APHA 2005). A Waters ionic chromatograph equipment, with a Pack A HC-4 column (6 × 150 mm, 10 μm), Dionex DSS conductivity detector and a mobile phase of HCO_3_^−^/CO_3_^2−^ buffer at 1.2 mL/min was used. Additionally, adsorbed ammonium on the bed surface was quantified from aqueous extraction of the compost following the Berthelot spectrophotometric method (Mulvaney 1996).

In phase VI, an extract of the compost was prepared in 96% ethanol at a 1:10 ratio to determine the presence of adsorbed toluene against a toluene standard, by the EPA 5021A method using a head-space gas chromatography technique in a Varian GC CP-3800 chromatograph with a FID detector.

### Statistical analysis

The comparison of the removal efficiency, elimination capacity, pH, ion concentration, and microbial population between the biofiltration stages and between sections were analyzed using Kruskal-Wallis and Wilcoxon tests for non-parametric data, because they have a non-normal distribution. The assumptions of these tests are that samples are independent random samples from their population and each data have similar variance (Smalheiser 2017). The tests were carried out in RStudio.

## Results and discussion

### Composting

The temperature of the compost did not increase above 24 °C, even though it was expected to reach 40 to 60 °C during the stages of organic matter decomposition and pathogen removal (Azim et al. 2018). This is due to the high content of recalcitrant carbon such as cellulose, hemicellulose, and lignin, which constituted the sugarcane bagasse (which was in a proportion of 60% of the total compost mixture) and the wood chips from the chicken manure (Haghdan et al. 2016). This high proportion of structuring agents during composting was important to increase porosity and airflow, and to avoid anaerobiosis which produces greenhouse gases such as methane (Sharma et al. 2017). Nevertheless, biological activity was achieved because a decrease in the percentage of organic matter (OM) was observed owing to its degradation to CO_2_ and humus. Furthermore, the microbial activity was significant at week 6 when the moisture was increased to 45% to accelerate compost degradation, demonstrating the importance of water for microbial metabolism.

Additionally, structuring agents increase organic carbon content, which decreases NH_3_ volatilization because it can be avoided if the initial C/N relation is between 35 and 25 (Bernal et al. 2009). In this work, C/N relation is 25. NH_3_ emission occurs because of high initial NH_4_^+^ concentration in manures. NH_4_^+^is deprotonated to form NH_3_, which is then released as gas (Bernal et al. 2009).

Moreover, the biodegradability coefficient (*Km*) raised to 0.7 indicating a good grade of stability of the material compared to other reports that showed a *Km* of 0.7 (Forero et al. 2018) and 0.9 (Vela-Aparicio et al. 2020). An increase of *Km* was expected due to the decomposition or decrease of organic matter percentage, indicating the degradability achieved during the composting process and the stability of the material in the presence of the microbial population (Haug 1993). In consequence, values close to 1.00 show a higher degree of OM degradation and thus stability.

### Characterization of compost as packing material

The porosity of the final compost was 40 ± 1% (Table 2), which is within the range that a biofilter bed should have (40% and 90%) to allow the growth of the microbial community and avoid pressure drops because it does not obstruct the air flow in the biofilter (Anet et al. 2013). Moreover, this value is close to the 43% as reported by Maestre et al. (2007) for activated sludge compost used to remove of 90% of toluene. Additionally, the density of 538 ± 4 g/L (Table 2) is lower than the one reported (753 g/L) by Vela-Aparicio et al. (2020) for poultry manure and sugarcane bagasse compost (1:1 ratio) used for the biofiltration of H_2_S and NH_3_. This was attributed to the increase in the proportion of sugarcane bagasse as a structuring agent from 50% by these authors to 60% to avoid compaction.

**Table 2:** Physical and chemical characteristics of compost of poultry manure and sugarcane bagasse as packing bed

Characteristics	Value
Density g/L	538 ± 4
pH	8.05 ± 0.01
Conductivity (μS/cm)	1758 ± 70
% Size particle distribution	
> 4.76 mm	14
4.76–2.38 mm	46
2.38–1.19 mm	31
1.19–0.59 mm	5
< 0.59 mm	4
Water retention capacity g H_2_O/ g compost	1.53 ± 0.08
Buffer capacity mol H^+^/kg compost	0.551 ± 0.04
% organic matter	76.91 ± 0.76
Porosity (%)	40 ± 1
CFU^a^ AOB/g	1.50 × 10^9^ ± 8.46 × 10^8^
CFU NOB/g	1.66 × 10^9^ ± 9.13 × 10^8^
CFU SOB/g	1.46 × 10^7^ ± 6.57 × 10^6^
CFU heterotrophs/g	1.47 × 10^9^ ± 8.18 × 10^8^

^a^*CFU* colony-forming units

Additionally, according to the authors (Barbusinski et al. 2017; Dorado et al. 2010), the pressure drop is generated by particle sizes smaller than 1 mm, and in this work, 91% of the particles were larger than 1.19 mm (Table 2). Besides, compared to Vela-Aparicio et al. 2020, in both studies the percentage of particle sizes larger than 2.38 mm was 60%, which is also favorable for biofiltration, since Vela-Aparicio et al. (2020) reported a removal efficiency of 99% for H_2_S and 95% for NH_3_ and in this work 96.9%, 68%, 79.1% for H_2_S, NH_3_, and toluene, respectively. Likewise, Das et al. (2019) obtained a yield greater than 99% for H_2_S using a compost bed of softwood bark, mixed garden waste, and organic fertilizers with a particle size between 2.8 and 5.6 mm. Furthermore, López et al. (2011) reported VOCs removal efficiencies between 45 and 90% in a biofilter of compost from kitchen and pruning waste (1.42:1 ratio), with particle size between 2 and 7 mm.

The pH 8.05 of the compost (Table 2) is optimal for biofiltration, considering that it should be between 6 and 8 to ensure good biological activity (Barbusinski et al. 2017). This value is also close to other composts previously used in biofiltration, such as mulch compost with pH 8.62, and with a removal efficiency higher than 90% for the reduction of odor units (Anet et al. 2013). Besides, the buffer capacity of 0.51 mol H^+^/Kg (Table 2) is higher than that reported by Vela-Aparicio et al. (2020), which was 0.37 mol H^+^/kg for manure-cane bagasse (1:1 ratio) compost. It is also higher than other types of manure compost reported by Costello and Sullivan (2014), whose buffer capacity ranged from 0.45 to 0.29 mol H^+^/kg. These results indicate the capacity to prevent bed acidification during the biological oxidation of H_2_S, which produces hydronium ions (H^+^).

The conductivity of 1758 μs/cm (Table 2) was higher than the conductivity of 1109 μs/cm for the residual sludge and wood chips compost (1:1 ratio), which reached a removal percentage of up to 100% in the biofiltration of butyric acid (Márquez et al. 2021). On the other hand, the organic matter of the compost in this work (76%) was comparable to 85.5% of the same compost reported by Márquez et al. (2021). These results indicate a high concentration of nutrients and ions such as phosphate ions (PO_4_^3−^), potassium (K^+^), sodium (Na^+^), magnesium (Mg^2+^), calcium (Ca^2+^), iron (Fe^2+^), zinc (Zn^2+^), and copper (Cu^2+^), which allow the maintenance of microbiological activity during the biofiltration process. However, further analysis of these anions is recommended. Regarding the water holding capacity (WHC), the value of 1.53 ± 0.08 g/g (Table 2) is comparable to other reports, like 1.2 g/g for compost of manure-cane bagasse (1:1 ratio) for the treatment of H_2_S and NH_3_ (Vela-Aparicio et al. 2020) and 1.74 g/g for compost of softwood bark, mixed yard waste, and organic fertilizers for the treatment of H_2_S (Das et al. 2019). This value indicates that the reduction in water evaporation of the biofilter would be lower, thus maintaining the moisture to preserve microbiological activity and removal efficiency (Dorado et al. 2010).

Finally, the bacterial populations of AOB, NOB, SOB, and heterotrophic bacteria were higher than 10^6^ CFU/g (Table 2), which is the expected value at the beginning of biofiltration, according to Delhoménie and Heitz (2005). Therefore, the high abundance of AOB and NOB indicated the capability of the compost to degrade NH_3_. Whereas the abundance of SOB and heterotrophic bacteria suggests the capability to degrade H_2_S and VOCs such as toluene, respectively.

### Pollutants removal efficiency and byproducts assessment

**Fig. 2:**
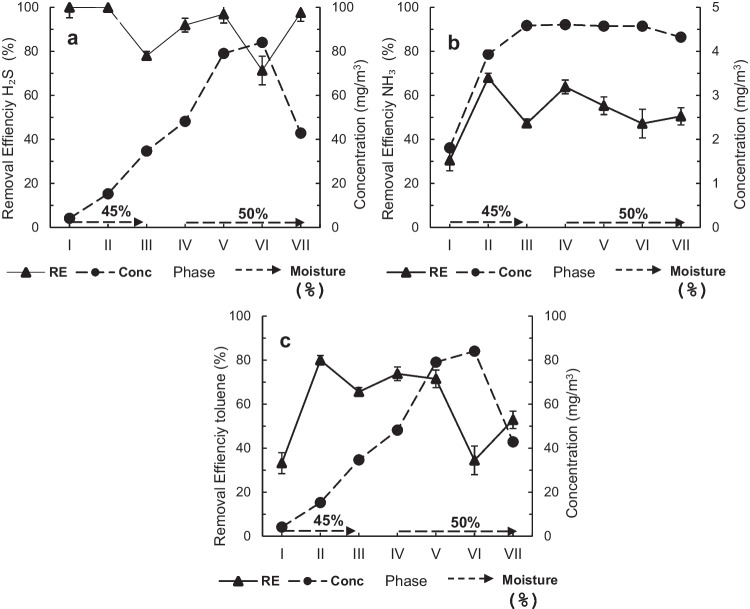
Removal efficiency (%RE) and inlet concentration (Conc) in biofiltration phase I to VII, at moisture 45% or 50% for (**a**) H_2_S, (**b**) NH_3_, and (**c**) toluene

**Fig. 3:**
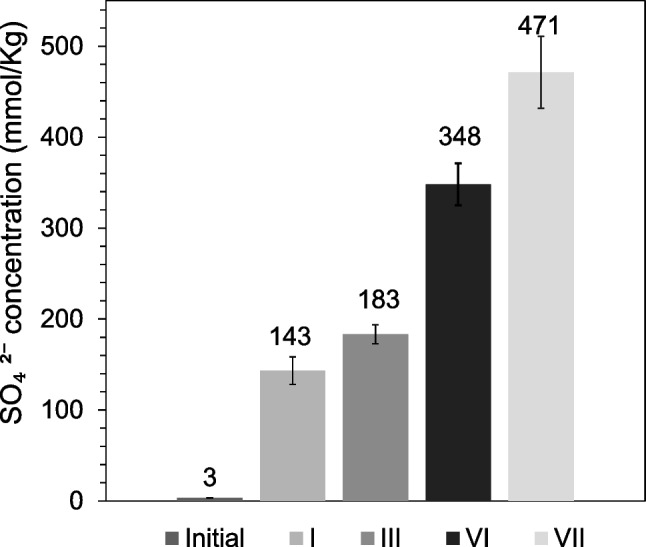
Concentration (mmol/Kg) of SO_4_^2−^ in phases: initial, I, III, VI, and VII

During phase I, the prototype biofilter unit was exposed to an H_2_S concentration of 4.2 mg/m^3^ while NH_3_ was 0.9 mg/m^3^ and toluene was 5.8 mg/m^3^ (Table 1). The H_2_S %RE from the beginning of the biofiltration was 100 ± 1% (Fig. 2(a), *Online Resource Table* *2*). This suggests that during the first days of phase I, H_2_S was rapidly removed owing to its diffusion towards the biofilm and physical adsorption on the packing material (Park et al. 2008). However, at the end of phase I, the removal was also due to oxidation by SOBs, since the sulfate (SO_4_^2−^) concentration increased to 143 mmol/kg, compared to 3 mmol/kg present in the compost before biofiltration (Fig. 3).

On the other hand, the prototype biofilter unit did not remove toluene or NH_3_ during the first 5 and 10 days of operation. This behavior is attributed to the fact that the microbial communities of AOB, NOB, and heterotrophic bacteria were in the acclimatization phase, where they were still developing metabolites to degrade these compounds (Knapp and Bromley-Challoner 2003). Furthermore, the toluene adsorption is low due to its hydrophobic nature. However, after these days, toluene and NH_3 _%RE increased to 33.2 ± 4.7% and 30 ± 5% (Fig. 2(b, c), *Online Resource Table* *2*), respectively, suggesting that the removal of these gases was due to microbiological biodegradation, and in the case of toluene due to its adsorption on the wet bed, which is low because of its hydrophobic nature (Ondarts et al. 2012). Similar results have been reported for toluene removal with a mixture of compost and activated carbon (Kumar et al. 2019), and with a biofilter of compost from green garden waste (Ondarts et al. 2012).

**Fig. 4:**
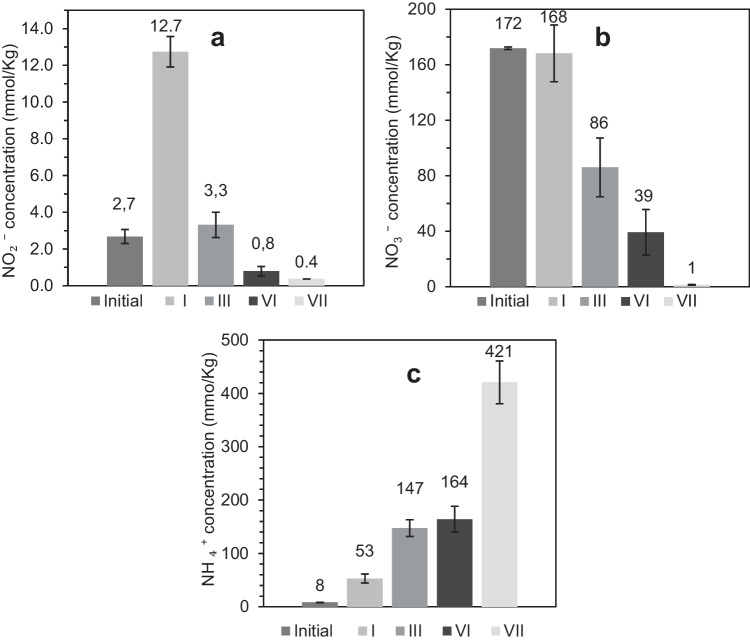
Concentration (mmol/kg) of (**a**) nitrite NO_2_^*−*^, (**b**) nitrate NO_3_^*−*^, and (**c**) ammonium NH_4_^+^ in phases: initial I, III, VI, and VII

Regarding NH_3_, part of this pollutant is oxidized to nitrite (NO_2_^−^) by AOB since the concentration of this ion increased from 2.7 mmol/kg (before biofiltration) to 12.7 mmol/kg at the end of phase I (Fig. 4(a)). On the contrary, no significant oxidation of nitrite (NO_2_^−^) to nitrate (NO_3_^−^) was observed by the NOB bacteria, as evidenced by the fact that the initial nitrate (NO_3_^−^) concentration was statistically equal to the concentration of phase I (Fig. 4(b)). This result is due to the low activity of NOBs during acclimatization and the possible inhibitory effect of H_2_S on these bacteria (Galera et al. 2008). Nevertheless, most of the NH_3_ was removed as ammonium ions (NH_4_^+^). which were formed by the contact of NH_3_ with the water of the wet bed. Consequently, the ammonium concentration (NH_4_^+^) increased from 8 mmol/kg (before biofiltration) to 53 mmol/kg (Fig. 4(c)).

During phase II, the H_2_S concentration was increased to 15.3 mg/m^3^, NH_3_ to 2.0 mg/m^3^, and toluene to 10.9 mg/m^3^ (Table 1). The H_2_S %RE remained at 100 ± 1%, indicating that the SOBs became more resistant to higher H_2_S loading rates after acclimatization (Fig. 2(a), *Online Resource Table* *2*). On the other hand, NH_3_ %RE increased to 68 ± 2% (Fig. 2(b), *Online Resource Table* *2*) and the toluene %RE to 80.0 ± 2.1% (Fig. 2(c), *Online Resource Table* *2*) because AOB and NOB improved their capacity to metabolize NH_3_ and heterotrophic bacteria to metabolize toluene. Later, in phase III, when the biofilters were exposed to a H_2_S concentration of 34.7 mg/m^3^, NH_3_ of 2.4 mg/m^3^, and toluene of 14.8 mg/m^3^ (Table 1), the H_2_S %RE decreased to 78.0 ± 2.2 (Fig. 2(a), *Online Resource Table* *2*), NH_3_ %RE decreased to 47 ± 3% (Fig. 2b, *Online Resource Table* *2*), and toluene %RE decreased to 65.6 ± 2.0 (Fig. 2(c), *Online Resource Table* *2*). These results were caused by the presence of dry areas with cracks and compaction, while the moisture was 45%, as these changes in the bed structure could generate differential flow and low oxygen diffusion (Maia et al. 2012). Consequently, neither the transfer of pollutants to the bed nor the microbial activity increased.

As a result of a decrease in oxygen diffusion, H_2_S oxidation decreased because sulfate concentration (SO_4_^2−^) only increased 40 mmol/kg from phase I (143 mmol/kg) to phase III (183 mmol/kg) (Fig. 3), in contrast to the increase in sulfate concentration between the start of biofiltration and phase I, which was higher (140 mmol/kg) (Fig. 3). Regarding NH_3_, low oxygen diffusion affected the oxidative activity of the AOB and NOB bacteria because the concentrations of both nitrite (NO_2_^−^) and nitrate (NO_3_^−^) were reduced from 168 to 86 mmol/kg and from 12.7 to 3.3 mmol/kg, respectively, compared to the concentration of phase I (Fig. 4(a, b)). This result indicates the process of denitrification, which is the reduction of nitrate or nitrite to nitric oxide, nitrous oxide, and nitrogen gas (Kuypers et al. 2018). However, the ammonium (NH_4_^+^) concentration increased from 53 to 147 mmol/kg (Fig. 4c); therefore, the removal in this phase was also due to adsorption on the bed.

In phase IV, the biofilter was exposed to a H_2_S concentration of 48.2 mg/m^3^, the NH_3_ concentration of 2.4 mg/m^3^, and toluene concentration of 16.4 mg/m^3^ (Table 1). The H_2_S %RE increased to 91.9 ± 2.0% (Fig. 2(a), *Online Resource Table* *2*), NH_3_ %RE increased to 64 ± 3% (Fig. 2(b), *Online Resource Table* *2*), and toluene %RE increased to 73.8 ± 3.1% (Fig. 3(c), *Online Resource Table* *2*) because the bed moisture was increased to 50% and oxygen flow was improved. The above results demonstrate the ability of the biofilter to recover from stress or transient conditions, such as the decrease in moisture that can occur during the treatment of malodours in a wastewater treatment plant. Furthermore, this result proves that NH_3_ adsorption increased with moisture because this compound is highly soluble in water, as reported by Liu et al. (2017) for a bed of a mixture of wood chips and compost in a 12:1 weight ratio. For toluene, despite being a hydrophobic compound and mixed with NH_3_ and H_2_S, the removal increases because there is more water available on the cell surface for enzymes to perform the highest load degradation of toluene (Beuger and Gostomski 2009). Furthermore, as toluene is consumed, the concentration gradient increases from the gas phase to the biofilm which enhances toluene removal by heterotrophic bacteria (Malhautier et al. 2005). Similar results of improve toluene removal with increasing moisture were reported by Sun et al. (2002) for a biofilter bed of compost and perlite but in comparison to this study, this report did not include volatile inorganic compounds such as NH_3_ and H_2_S, highlighting the novelty of these results. 

During phase V, the biofilter was exposed to a transient condition with a high concentration of H_2_S (79.1 mg/m^3^), which is less frequent in the El Salitre WWTP. On the other hand, the NH_3_ concentration was 2.4 mg/m^3^ and toluene 22.3 mg/m^3^. H_2_S %RE continued to increase to 96.9 ± 1.2% (Fig. 2(a), *Online Resource Table* *2*) and toluene %RE was statistically equal to phase IV of 71.5 ± 4.0% (Fig. 2(c), *Online Resource Table* *2*). A similar result was previously reported by Vela-Aparicio et al. (2020) with compost of manure-cane bagasse (1:1 ratio), for the same concentration of H_2_S (79.1 mg/m^3^) and they obtained a %RE of 100% while NH_3_ was 1–2.6 mg/m^3^ but without toluene flow. This demonstrates the biofilter resistance to a shock concentration of H_2_S in a mixed stream with toluene which is a recalcitrant compound that is not studied in the Vela-Aparicio et al. (2020) report.

However, during this phase, NH_3_ %RE decreased to 55 ± 4% (Fig. 2(b), *Online Resource Table* *2*) due to the inhibition of AOB and NOB by H_2_S (Jiang and Tay 2010; Yoon et al. 2002). Later, during phase VI, the prototype biofilter unit was exposed to concentration peaks of both H_2_S and toluene, H_2_S concentration was 84.1 mg/m^3^, toluene was increased to 40.9 mg/m^3^, while NH_3_ concentration remained constant. In consequence, H_2_S %RE decreased to 71.4 ± 4.3% (Fig. 2(a), *Online Resource Table* *2*), NH_3_ %RE decreased to 47 ± 5% (Fig. 2(b), *Online Resource Table* *2*), and toluene %RE decrease to 34.5 ± 6.5% (Fig. 2(c), Table 2). Therefore, these results indicate that this toluene concentration may be toxic for SOBs, AOB NOBs, and even heterotrophic bacteria that are not able to degrade the toluene load completely. A similar behavior was reported by Lebrero et al. (2010) during the biofiltration of H_2_S and toluene, using a mixture of compost (75%) and perlite (25%) as bed, when toluene concentration increased from 0.1 to 0.9 mg/m^3^ and H_2_S from 0.5 to 3.0 mg/m^3^, H_2_S removal efficiency decreased from 98.0 to 91.9% and toluene from 98.4 to 69.5%.

Likewise, denitrification was also observed during this phase, as nitrite (NO_2_^−^) and nitrate (NO_3_) were reduced to 0.8 mmol/kg and 39 mmol/kg, respectively (Fig. 4(a, b)), resulting in the formation of NO_2_ at a concentration of 0.337 ppm. Nevertheless, this emission is very close to the environmental level of 0.321 ppm (Hu 2021), and therefore does not represent a dangerous pollutant stream of the biofiltration technology. On the other hand, ammonium concentration increased to 164 mmol/kg, indicating that adsorption was the main mechanism of NH_3_ removal. In the case of H_2_S, despite the decrease in removal, there was some SOB activity as the sulphate concentration increased to 300 mg/kg (Fig. 3). For toluene, adsorption on the surface was negligible, again indicating a biological mechanism for its removal.

In phase VII, the biofilter was exposed to pollutant-free air for 7 days and then the pollutant concentrations were increased as indicated in Table 1. Under these conditions, SOB recovered its activity because H_2_S %RE increased to 97.0 ± 0.9% (Fig. 2(a), *Online Resource Table* *2*) and sulfate concentration (SO_4_^2^) increased to 471 mmol/kg (Fig. 3). Heterotrophic bacteria also improved the activity because toluene %RE increased to 52.9 ± 3.9% (Fig. 2(c), *Online Resource Table* *2*). However, AOB and NOB did not recover their activity since nitrite (NO_2_^−^) and nitrate (NO_3_^−^) concentrations were negligible (Fig. 4(a, b)). However, NH_3_ %RE increased to 50 ± 5% due to adsorption as NH_4_^+^ which increased its concentration to 421 mmol/kg. These results demonstrate the ability of the biofiltration system to adapt to shock loads and stress situations that may occur in a WWTP. Furthermore, this indicates the importance of resting periods/shutdown to recover from transient conditions that can be toxic to microorganisms, as previously reported by Vela-Aparicio et al. (2021) for H_2_S and NH_3_ biofiltration in a bed of manure and sugarcane bagasse (1:1 ratio).

H_2_S %RE increased when moisture was increased in phase III, showing the high biological dependence mechanism of degradation. However, in phase VI, it was observed that toluene concentrations above 40.9 mg/m^3^ had a toxic effect on the SOB, resulting in a decrease of H_2_S %RE from 100 to 71.4 ± 4.3%. During phase VII, when H_2_S concentration decreased to 42.9 mg/m^3^, %RE increased to 97.0 ± 0.9% which is almost the same %RE obtained at a similar concentration in the phase IV (48.2 mg/m^3^). However, H_2_S %RE increased to 52.9 ± 3.9% when toluene concentration was decreased to 15.1 mg/m^3^, but it was lower than the %RE obtained in phase III and IV (%RE 65.6 ± 2.0% and 73.8 ± 3.1%, respectively) when toluene concentration was around 15 mg/m^3^ (14.8 ± 1.4 mg/m^3^ for phase III;16.4 ± 2.7 mg/m^3^ for phase IV). This result may indicate a greater negative effect of H_2_S on heterotrophic bacteria.

Finally, the %RE of H_2_S was comparable with other reports with similar loading rates and using compost as a packing bed, whereas the %RE of NH_3_ was lower (Table 3). However, these authors treated only VICs whereas in this work toluene is included in the pollutant stream as a volatile organic compound. Besides that, the emission of NH_3_ is 10 times lower than the exposure limit of 18 mg/m^3^ required by the United States Occupational Health Administration (OSHA; Occupational Safety and Health Administration 2020). On the other hand, compared to Galera et al. (2008), who also used compost and treated these three compounds, it was not necessary to seed the bed with pure strains, which makes this technology more economical and accessible. The above indicates the potential use of materials that are easily obtained in Colombia such as manure and sugarcane bagasse for use in the simultaneous treatment of H_2_S and NH_3_, as well as toluene. This is important in the operation of a WWTP, as the latter volatile compound has a different chemical nature, is hydrophobic, and is not generally reported.

**Table 3:** Elimination capacity (EC) and percentage removal efficiency (%RE) of biofilter with compost as packing bed for the removal of H_2_S and/or NH_3_ with a similar loading rate (LR) to those in this study. Empty bed residence time (EBRT) and flow rate (Q) are also shown for each study

Compound/s	Bed	Parameters	EC RE	Author
H_2_S NH_3_	Activated sludge + PVC + compost	EBRT: 15 s; Q: 2.16 m^3^/h LR H_2_S: 3.9 g/m^3^ h; TC NH_3_: 0.5–1.8 g/m^3^ h	H_2_S EC: 3,6g/m^3^h; %RE: 92% NH_3_ EC: 1.7 g/m^3^ h %RE 99.5%	Alinezhad et al. (2019)
H_2_S	Compost and sand (1:4)	EBRT: 10 min; Q: 6 m^3^/h LR H_2_S: 0.212 g/m^3^	EC: 0.108 g/m^3^ h %RE 50.9%	Yuan et al. (2019)
H_2_S NH_3_	Compost chicken manure and sugarcane bagasse (5:5)	EBRT: 60 min; Q: 0.39 m^3^/h LR H_2_S: 4.7 g/m^3^ h LR NH_3_ 1.2 g/m^3^ h	H_2_S EC: 3.6 g/m^3^ h; %RE: 100% NH_3_ EC:1.7g/m^3^ h %RE 99,5%	Vela-Aparicio et al. (2020)
H_2_S NH_3_ toluene	Compost chicken manure and sugarcane bagasse (4:6)	EBRT: 60 min; Q: 0.39 m^3^/h LR H_2_S: 4.7 g/m^3^ h LR NH_3_ 1.2 g/m^3^ h LR toluene: 1.32 g/m^3^ h	H_2_S EC: 4.7 g/m^3^ h; %RE: 96.9% NH_3_ EC: 1.7 g/m^3^ h; %RE: 68% Toluene EC: 0.97 g/m^3^ h %RE 71.5%	This work

### Pollutant removal in each section

**Fig. 5:**
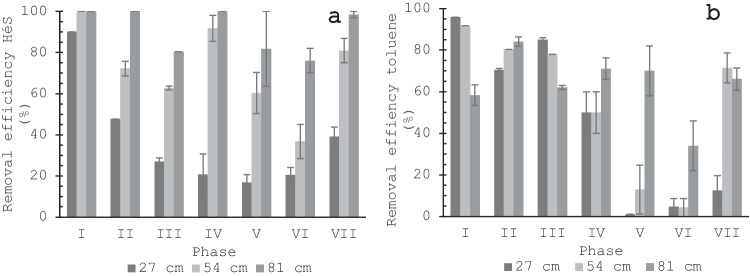
Removal efficiency (%) of (**a**) H_2_S, (**b**) toluene depending on the height of the biofilter: low section (27 cm), middle section (54 cm), upper section (81 cm) depending on the biofiltration phase

For the purpose of discussion, the lower section is located at 27 cm from the inlet, the middle section at 54 cm and the upper section at 81 cm. Most of the H_2_S %RE (90%) during phase I was performed in the lower section, and during phases II, III, and IV in the middle section (60–90%), due to the increase of the loading rate. However, during phase VI, when the system was exposed to high loading rates of H_2_S (4.97 g/m^3^ h) and toluene (2.42 g/m^3^ h), 76% of the H_2_S was removed in the upper section (81 cm) (Fig. 5(a, b)). In the case of toluene, 70-90% of the gas was removed in the lower section during phases I, II and III, and 34-73% of the gas was removed in the upper section during the higher loading phases IV, V and VII. Thus, at higher loading rates, the majority of the %RE occurs in the upper section, indicating that the constructed height was suitable for removal of the contaminants under transient conditions. Furthermore, this result indicates that the EBRT of 60 s is suitable for the removal of toluene and H_2_S. On the other hand, NH_3_ removal across the biofilter varied within sections because the ascending gas stream caused the desorption of NH_4_^+^ previously adsorbed. This explained why the maximum removal of NH_3_ was 68%.

### Microbiological, pH, and moisture analysis

The abundance of SOB, NOB, AOB, and heterotrophic bacteria decreased in phase I, compared to the initial compost due to the toxic effect of H_2_S (Table 4). However, in the later phases, the increase in gas concentration did not affect the bacteria population, as it was statistically equal (*p* > 0.05), in all phases (I to VII) and between sections, according to the Kruskal-Wallis test. These results suggest that the bacteria were acclimatized to carry out the degradation after phase I. Then, during phase II, the removal of all contaminants increased without decreasing the bacterial population. During phase III, there were no significant differences (*p* < 0.05) between the populations in comparison with phase I, despite the %RE decrease. The above results suggest that the production and activity of the enzymes which oxidize H_2_S, NH_3_, and toluene are determined by the amount of substrate or pollutants (Engelking 2013). Consequently, the microbiological activity decreased because the transfer of gases to the biofilm was affected due to the low moisture. Finally, in phase VI, the high concentration of toluene and H_2_S could have inhibited these enzymes without affecting the bacterial population. Therefore, during phase VII, the population was the same and the loss of microbiological activity may have been due to reduced gene expression of degradative enzymes (Guieysse et al. 2008).

**Table 4:** Microbial population count (log CFU/g) in the compost bed of sugarcane bagasse and chicken manure (ratio 6:4), in the different stages of biofiltration

Bacteria	Initial	Phase I	Phase III	Phase VI	Phase VII
Heterotrophic	9.2	8.0^**a,b**^	7.6^**b**^	7.9^**b**^	7.6^**b**^
NOB	9.2	7.5^**a,b**^	7.8^**b**^	7.6^**b**^	7.1^**b**^
AOB	9.2	7.2^**a,b**^	7.2^**b**^	8.1^**b**^	7.7^**b**^
SOB	7.2	6.6^**b**^	7.8^**b**^	7.4^**b**^	6.8^**b**^

^a^Statistically significant different to initial population

^b^Not statistically significant different to population of previous phases

Similar behavior was also reported by Lebrero et al. (2010) for a bed of compost (75%) and perlite (25%). This biofilter showed no change in the microbial community when the %RE decreased to 10% by increasing toluene concentration from 0.1 to 0.9 mg/m^3^ and increasing H_2_S concentration from 0.5 to 30 mg/m^3^. Likewise, during biofiltration of H_2_S, NH_3_ and toluene with an inoculated cork bed, no change in the community was observed either, even when the NH_3_ %RE decreased from 95 to 50% and toluene %RE decreased from 99 to 10% (Park et al. 2008). On the other hand, the differences between the change in the population compared to other authors compared to other authors, such as Vela-Aparicio et al. (2022), who observed an increase in the AOB population, may be due to the type of microbial community installed in the bed at the beginning of the biofiltration, as well as the effect of toluene which could have affected the growth of the bacterial population. However, in this work, the low fluctuation in the microbial community indicates the long viability of the biofilter for industrial use.

On the other hand, the pH of the system was statistically equal in phases I, III, and VI, and between sections, with an average value of 7.8, which demonstrates the good buffering capacity of the system, which was 0.51 mol H^+^/kg of compost. The low pH variation is also due to the production and consumption of hydronium H^+^ ions in three simultaneous reactions. On the one hand, the oxidation of H_2_S produces sulfate (SO_4_^2−^) and hydronium H^+^ ions, while nitrate denitrification (NO_3_^−^) consumes hydronium H^+^ ions and NH_3_ adsorption produces hydroxyl OH^−^ ions, which increase the pH. Moreover, in stage VII the pH decreases to 6.5, possibly because the concentration of nitrite (NO_2_^−^) and nitrate (NO_3_^−^) is negligible and therefore the production of hydronium H^+^ ions prevails in the oxidation of H_2_S. The tendency to decrease pH was also reported by Galera et al. (2008), who evaluated the simultaneous removal of H_2_S, NH_3_, and toluene in a bed of rock wool and compost, as well as by Hou et al. (2016) with a decrease from 8.0 to 6.8 in the removal of H_2_S and NH_3_, due to the high production of sulfate (SO_4_^2−^) in a compost bed, straw and wood chips (ratio 70:15:15). Finally, also by Vela-Aparicio et al. (2022) with a reduction of pH from 7.5 to 6.5. However, according to these results, the pH remained in the range of 6–8, which is optimal for the functioning of SOB, AOB, NOB, and heterotrophic bacteria.

Biofiltration of H_2_S is known to lower the pH of the biofilter and can affect gas removal. However, the results of this study demonstrate that the packing material used is not affected by the oxidation of H_2_S and maintains a stable pH level. This is a significant operational advantage as pH control in a biofilter can often be challenging. This prototype biofilter unit is therefore suitable for the effective removal of odours in a WWTP.

In addition, one of the most critical operational limitations of a biofilter is the moisture content of the packing material. In this work, moisture increasing from 45 to 50% improved the %RE of all three compounds studied, even though toluene is hydrophobic. This increase was due to the importance of water for biofilm formation and the microbiological activity of bacteria. Previously, the increase in toluene %RE due to moisture increasing has also been reported in 7:3 compost and perlite bed biofilters (Sun et al. 2002) and in soil bed (Badilla et al. 2011). However, it is recommended to keep the moisture level below 55%, as higher levels can also cause compaction, denitrification, and pressure drop, as was reported by Liu et al. (2017) when using a packing material of wood chips and compost in a 12:1 weight ratio. Additionally, it is important to note that no pressure drop was observed in this work because the inlet and outlet flow were the same during the whole experiment.

### Pathways of biodegradation

Sulfur-oxidizing bacteria (SOB) oxidize H_2_S to elemental sulfur (S) (Eq. 5), by the enzyme adenosine phosphosulfate reductase (Pokorna and Zabranska 2015). Subsequently, in excess of O_2_, elemental sulfur (S) is oxidized to sulfate (SO_4_^2−^) in the form of sulfuric acid (H_2_SO_4_) (Eq. 6) which overall reaction is shown in equation (Eq. 7). If there is a high hydrogen sulfide (H_2_S) load in biofiltration systems and the O_2_/H_2_S ratio is less than 1, it will lead to incomplete oxidation only to elemental sulfur (S) (Eq. 5). This can affect the operation of the biofilter by generating pressure drops (Lin et al. 2018). In this work, sulfate (SO_4_^2−^) was quantified in the bed during all phases, but an additional analysis may be needed to quantify a possible production of elemental sulfur (S).

5 $$2{\mathrm{H}}_2\mathrm{S}+{\mathrm{O}}_2\to 2{\mathrm{S}}^0+2{\mathrm{H}}_2\mathrm{O}$$

6 $$2{\mathrm{S}}^0+2{\mathrm{H}}_2\mathrm{O}+3{\mathrm{O}}_2\to 4{\mathrm{H}}^{+}+2{{\mathrm{S}\mathrm{O}}_4}^{2-}$$

7 $${\mathrm{H}}_2\mathrm{S}+2{\mathrm{O}}_2\to 2{\mathrm{H}}^{+}+{{\mathrm{SO}}_4}^{2-}$$

Regarding ammonia (NH_3_), it is first adsorbed on the wet surface of the packing material and produces ammonium (NH_4_^+^). Later ammonium (NH_4_^+^) is oxidized to nitrite (NO^2−^) by ammonia-oxidizing bacteria (AOB) according to Eq. 8. To do this, first the ammonium monooxygenase (AMO) enzymes catalyze the oxidation of ammonium (NH_4_^+^) to hydroxylamine (NH_2_OH) as shown in Eq. 9. Then, the hydroxylamine oxidoreductase (HAO) enzyme catalyzes the oxidation of hydroxylamine (NH_2_OH) to nitric oxide (NO) and later to nitrite (NO_2_^−^) according to Eqs. 10 and 11, respectively (Kuypers et al. 2018).

8 $$2{\mathrm{NH}}_4^{+}+3{\mathrm{O}}_2\to 2{\mathrm{NO}}_2^{-}+4{\mathrm{H}}^{+}+2{\mathrm{H}}_2\mathrm{O}$$

9 $${\mathrm{NH}}_4^{+}+2{\mathrm{e}}^{-}+{\mathrm{O}}_2+{\mathrm{H}}^{+}\to {\mathrm{NH}}_2\mathrm{O}\mathrm{H}+{\mathrm{H}}_2\mathrm{O}$$

10 $${\mathrm{NH}}_2\mathrm{OH}\to \mathrm{NO}+3{\mathrm{e}}^{-}+3{\mathrm{H}}^{+}$$

11 $$\mathrm{NO}+{\mathrm{H}}_2\mathrm{O}\to {\mathrm{NO}}_2^{-}+{\mathrm{e}}^{-}+2{\mathrm{H}}^{+}$$

Secondly, nitrite (NO_2_^−^) is oxidized to nitrate (NO_3_^−^) (Eq. 12) by nitrite oxidizing bacteria (NOB) in a reaction is catalyzed by the enzyme nitrite oxidoreductase (NRX) (Kuypers et al. 2018). As it was shown previously, nitrite (NO_2_^−^) and nitrate (NO_3_^−^) were monitored but the main mechanism of ammonia (NH_3_) is by adsorption and formation of ammonium (NH_4_^+^).

12 $$2{\mathrm{NO}}_2^{-}+{\mathrm{O}}_2\to 2{{\mathrm{NO}}_3}^{+}$$

In the case of toluene, there are different possible degradation pathways. More research must be done to identify them. Some of the reported pathways are Tod Dioxygenase-mediated pathway, TOM Toluene-2-monooxygenase pathway, Tbu Toluene-3-monooxygenase pathway, TMO Toluene-4-monooxygenase pathway, TOL Toluene methyl monooxygenase pathway (Muccee et al. 2019). But in general, VOCs are oxidized to CO_2_ (Eq. 13) (Xie et al. 2009).

13 $${\mathrm{CO}\mathrm{V}}_{\mathrm{s}}\to {\mathrm{CO}}_2+{\mathrm{H}}_2\mathrm{O}$$

### Long-term operation

For long-term operation, it is necessary to maintain the moisture level of the bed, which can be achieved by using a humidifier that releases pressurized water as steam. The humidifier is coupled to the polluting air stream and in consequence the inlet flow is adjusted with its stream. For example, Kumar et al. (2019) reported the use of a humidifier for the removal of toluene using a mixed bed of compost and activated carbon. On the other hand, it is also possible to adjust moisture by direct irrigation on the top of the biofilter or combine both technologies, as reported in industrial use by Omri et al. (2013) for the treatment of H_2_S in the Charguia WWTP, Tunis, Tunisia.

For biofilter installation in a WWTP, it is necessary to close the area and determine the volume of contaminated air with the dimensions of the structure. Then, according to a ventilation rate (*Vr*), which is the number of times per hour that the volume of contaminated air contained within the confined space is extracted, it is possible to calculate the industrial flow rate using the following equation (Eq. 14):

14 $${Q}_{\mathrm{gas}}={V}_{\mathrm{polluted}\ \mathrm{air}}\times Vr$$

where, *Q*_gas_: flow rate of polluted air (m^3^/h); *V*_polluted air_: polluted air volume (m^3^); and *Vr*: ventilation rate (h^−1^).

The biofilter volume of an industrial application is calculated using this flow rate and the set EBRT of 60 s used in this work. A fan can be used to extract the air that is captured with tubing made of fiberglass-reinforced plastic to avoid corrosion with H_2_S and that connects to the biofilter to be treated (Hort et al. 2013). Subsequently, the air stream is directed to the biofilter to be treated. Additionally, it is important to measure the gas inlet flow, moisture content and pH of the bed and perform periodic sampling of the bed for analysis of metabolites and microbial population.

Concerning the biofilter packing material, compost lifespan is approximately 2 to 5 years (Dorado et al. 2010). When it has lost its useful life, the compost bed can be used as a fertilizer or soil conditioner depending on the concentration of heavy metals, microbiological pathogens, and in concordance to regulation of each country. In addition, it can be used in forest plantations, soil recovery, raw material of fertilizers, remediation of contaminated soils, raw material for manufacturing construction materials, stabilization of road network slopes, operations in landfills, revegetation, landscaping, or energy recovery (Ministerio de Vivienda, Ciudad y Territorio de Colombia 2014).

In general, this work has practical applications for the set up of the compost. Conditions of composting manure with wood chips and sugar cane bagasse, specifically moisture and particle size are crucial to obtain a desired material for biofilters, because its physical and microbiological characteristics demonstrate its use. Furthermore, the compost has an intrinsic microbial population which has economic benefits because it is not necessary to inoculate the bed in a pilot or industrial use and the addition of nutrients or solutions was not necessary, which has economic benefits in an industrial operation.

In addition, considering concentration fluctuation according to tropical seasonal weather in Colombia (dry and rainy season), the biofilter should be installed during the rainy weather season, which is characterized by low concentrations; therefore, the operation of the biofilter in this season will serve as acclimatization time for the bacteria in the bed. Afterwards, when emissions are higher during the dry season, the system will be acclimated, and odor removal will be effective. Besides, it is expected that this study will contribute to the formulation of laboratory studies which can also evaluate genetics of microbial population, and fluctuation of other operational parameters like EBRT, lower and higher moisture content. Furthermore, it will contribute to semi-pilot, pilot, and industrial biofiltration uses in countries with high production of manure and sugarcane bagasse waste.

## Conclusions

In this study, it was possible to construct a biofiltration prototype biofilter unit with a compost bed of sugarcane bagasse and chicken manure (60:40 ratio) which are economical materials and easy to obtain in Colombia, which demonstrates the suitability of the biofiltration technology to treat offensive odors. This prototype biofilter unit removed volatile inorganic compounds like H_2_S and NH_3_, and volatile organic compounds like toluene, simultaneously and in similar concentrations to those found at the El Salitre WWTP. It was found that NH_3_ did not negatively affect the degradation of H_2_S or toluene, considering that H_2_S is the compound that is emitted in the highest concentration and that contributes the most to the offensive odor. Meanwhile, H_2_S inhibited the microbiological degradation of NH_3_; and toluene negatively affected the removal of H_2_S when the concentration was higher than 40.9 mg/m^3^, which corresponds to a shock concentration of the WWTP. However, the biofilter recovered its capacity from this transient condition without adding nutrients or solutions that increase costs in an industrial operation.

H_2_S and toluene removal was due to bed adsorption and microbiological oxidation. It is also suggested that toluene removal was due to the same mechanisms of H_2_S, yet CO_2_ monitoring is recommended to ensure toluene oxidation. NH_3_ removal was mainly due to adsorption on the bed as ammonium and by denitrification. However, in this process, NH_3_ and N_2_O gasses were not emitted in polluting concentrations. Likewise, based on these mechanisms, the pH and microbial population did not fluctuate significantly during the operation of the prototype biofilter unit or even during transient conditions of high pollutant loading rate. Finally, bed moisture is one of the factors that most influence the removal efficiency of the biofilter. Therefore, it is recommended to use moisture greater than 45% and less than 55% for the maintenance of the biofilter media, which avoids a differential flow of oxygen and decreases denitrification.

## Supplementary information

ESM 1 Fig. S1 NH_3_ and VOCs concentrations (ppm) in relation to H_2_S concentration (ppm) found at El Salitre WWTP. Fig. S2 Reactor for a composting mix of sugar cane bagasse and manure. Fig. S3 Gas generation system assembly. Fig. S4 Biofilter assembly. Fig. S5 Calibration curve Na_2_S concentration (mg/L) added to HCl to obtain a desire concentration of H_2_S gas stream (ppm). Table 1 Most abundant organic compounds of the pre-treatment zone at El Salitre WWTP. Table 2 Removal efficiency (%) for H_2_S, NH_3_ and toluene in the different phases (DOCX 727 kb)

## Data Availability

The authors declare that all relevant data that support the findings of this study are available within the article. Additional data are available from the corresponding author upon reasonable request.

## References

[CR1] Alinezhad E, Haghighi M, Rahmani F, Keshizadeh H, Abdi M, Naddafi K (2019) Technical and economic investigation of chemical scrubber and bio-filtration in removal of H2S and NH3 from wastewater treatment plant. J Environ Manage 241:32–43. 10.1016/j.jenvman.2019.04.003 30981141 10.1016/j.jenvman.2019.04.003

[CR2] Anet B, Couriol C, Lendormi T, Amrane A, Le Cloirec P, Cogny G, Fillières R (2013) Characterization and selection of packing materials for biofiltration of rendering odourous emissions. Water Air Soil Pollut 224:1–13. 10.1007/s11270-013-1622-1

[CR3] APHA (2005) Standard methods for the examination of water and wastewater, 21st edn. American Public Health Association, Washington, DC

[CR4] Azim K, Soudi B, Boukhari S, Perissol C, Roussos S, Thami Alami I (2018) Composting parameters and compost quality: a literature review. Org Agric 8:141–158. 10.1007/s13165-017-0180-z

[CR5] Badilla DB, Gostomski PA, Dalida MLP (2011) Influence of water content on biofiltration performance. ASEAN J Chem Eng 10(2):31–39. 10.22146/ajche.50087

[CR6] Barbusinski K, Kalemba K, Kasperczyk D, Urbaniec K, Kozik V (2017) Biological methods for odor treatment—a review. J Clean Prod 152:223–241. 10.1016/j.jclepro.2017.03.093

[CR7] Bernal MP, Alburquerque JA, Moral R (2009) Bioresource technology composting of animal manures and chemical criteria for compost maturity assessment a review. Bioresour Technol 100:5444–5453. 10.1016/j.biortech.2008.11.027 19119002 10.1016/j.biortech.2008.11.027

[CR8] Beuger AL, Gostomski PA (2009) Development of a biofilter with water content control for research purposes. Chem Eng J 151:89–96. 10.1016/j.cej.2009.01.045

[CR9] Brancher M, Griffiths KD, Franco D, de Melo Lisboa H (2017) A review of odour impact criteria in selected countries around the world. Chemosphere 168:1531–1570. 10.1016/j.chemosphere.2016.11.160 27939667 10.1016/j.chemosphere.2016.11.160

[CR10] CEN (2007) CEN - EN 13040 Soil improvers and growing media -sample preparation for chemical and physical tests, determination of dry matter content, moisture content and laboratory compacted bulk density.

[CR11] Cha LMJ, Cha WS, Lee JH (1999) Removal of organo-sulphur odour compounds by Thiobacillus novellus SRM, sulphur-oxidizing microorganisms. Process Biochem 34:659–665. 10.1016/S0032-9592(98)00139-3

[CR12] Earthgreen Colombia. (2023). “Ficha Técnica SAC-100”. https://www.earthgreen.com.co/sistemas-de-compostaje Accessed 06 Jan 2023

[CR13] Costello RC, Sullivan DM (2014) Determining the pH buffering capacity of compost via titration with dilute sulfuric acid. Waste Biomass Valorization 5:505–513. 10.1007/s12649-013-9279-y

[CR14] Das J, Rene ER, Dupont C, Dufourny A, Blin J, van Hullebusch ED (2019) Performance of a compost and biochar packed biofilter for gas-phase hydrogen sulfide removal. Bioresour Technol 273:581–591. 10.1016/j.biortech.2018.11.052 30476867 10.1016/j.biortech.2018.11.052

[CR15] Delhoménie MC, Heitz M (2005) Biofiltration of air: a review. Crit Rev Biotechnol 25:53–72. 10.1080/07388550590935814 15999852 10.1080/07388550590935814

[CR16] Dorado AD, Lafuente FJ, Gabriel D, Gamisans X (2010) A comparative study based on physical characteristics of suitable packing materials in biofiltration. Environ Technol 31:193–204. 10.1080/09593330903426687 20391804 10.1080/09593330903426687

[CR17] Engelking LR (2013) Chapter 5—properties of enzymes. In: Textbook of veterinary physiological chemistry, 3rd edn. Academic Press, pp 26–31. 10.1016/B978-0-12-391909-0.50005-0

[CR18] Forero DF, Peña CE, Acevedo P, Hernández MA, Cabeza IO (2018) Biofiltration of acetic acid vapours using filtering bed compost from poultry manure—pruning residues—rice husks. Chem Eng Trans 64:511–516. 10.3303/CET1864086

[CR19] Galera MM, Cho E, Tuuguu E, Park S, Lee C, Chung W (2008) Effects of pollutant concentration ratio on the simultaneous removal of NH_3_, H_2_S and toluene gases using rock wool-compost biofilter. J Hazard Mater 152:624–631. 10.1016/j.jhazmat.2007.07.025 17714863 10.1016/j.jhazmat.2007.07.025

[CR20] Guieysse B, Hort C, Platel V, Munoz R, Ondarts M, Revah S (2008) Biological treatment of indoor air for VOC removal: potential and challenges. Biotechnol Adv 26:398–410. 10.1016/j.biotechadv.2008.03.005 18547770 10.1016/j.biotechadv.2008.03.005

[CR21] Haghdan S, Renneckar S, Smith GD (2016) Sources of Lignin. In: Lignin in Polymer Composites. Elsevier Inc, pp 4–5. 10.1016/B978-0-323-35565-0.00001-1

[CR22] Haug RT (1993) The practical handbook of compost engineering. Lewis Publishers. 10.1201/9780203736234

[CR23] Honeywell (2015) MultiRAE series. User’s guide. https://www.raespain.com/es/documents/multirae2_usersguide_rev_h_a4_es.pdf. Accessed 22 May 2024

[CR24] Hort C, Gracy S, Platel V, Moynault L (2013) A comparative study of two composts as filter media for the removal of gaseous reduced sulfur compounds (RSCs) by biofiltration: application at industrial scale. Waste Manag 33(1):18–25. 10.1016/j.wasman.2012.09.009 23036720 10.1016/j.wasman.2012.09.009

[CR25] Hou J, Li M, Xia T, Hao Y, Ding J (2016) Simultaneous removal of ammonia and hydrogen sulfide gases using biofilter media from the biodehydration stage and curing stage of composting. Environ Sci Pollut Res 23:20628–20636. 10.1007/s11356-016-7238-4 10.1007/s11356-016-7238-4 27464668

[CR26] Hu SM (2021) Chapter 8 - Trace gas measurements using cavity ring-down spectroscopy. In: Chen W, Venables DS, Sigrist MW (eds) Advances in spectroscopic monitoring of the atmosphere. Elsevier, pp 412–441. 10.1016/B978-0-12-815014-6.00002-6

[CR27] ICONTEC (2011) Agricultural industry products. organic products used as fertilizers and soil amendments. NTC 5167:2011

[CR28] Instituto Geográfico Agustín Codazzi (2006) Soil laboratory analytical methods, 6th edn. IGAC, Colombia

[CR29] Iranpour R, Cox HHJ, Deshusses MA, Schroeder ED (2005) Literature review of air pollution control biofilters and biotrickling filters for odor and volatile organic compound removal. Environ Prog 24:254–267. 10.1002/ep.10077

[CR30] Jiang G, Melder D, Keller J, Yuan Z (2017) Odor emissions from domestic wastewater: a review. Crit Rev Environ Sci Technol 47:1581–1611. 10.1080/10643389.2017.1386952

[CR31] Jiang X, Tay JH (2010) Microbial community structures in a horizontal biotrickling filter degrading H_2_S and NH_3_. Bioresour Technol 101:1635–1641. 10.1016/j.biortech.2009.09.074 19837581 10.1016/j.biortech.2009.09.074

[CR32] Kim IS, Ivanov VN (2000) Detection of nitrifying bacteria in activated sludge by fluorescent in situ hybridization and fluorescence spectrometry. World J Microbiol Biotechnol 16:425–430. 10.1023/A:1008949821236

[CR33] Knapp JS, Bromley-Challoner KCA (2003) Recalcitrant organic compounds (chemical oxygen demand sources) in biologically treated pulp and paper mill effluents: their fate and environmental impact in receiving waters. The Handbook of Water and Wastewater Microbiology. Elsevier, p 566. 10.2175/106143098x123705

[CR34] Kumar M, Shekher B, Kim K, Prasad R, Rene ER, López ME, Rai BN, Singh H, Prasad D, Sharan R (2019) Bioresource Technology Performance of a biofilter with compost and activated carbon based packing material for gas-phase toluene removal under extremely high loading rates. Bioresour Technol 285:121317. 10.1016/j.biortech.2019.121317 30979643 10.1016/j.biortech.2019.121317

[CR35] Kuypers MMM, Marchant HK, Kartal B (2018) The microbial nitrogen-cycling network. Nat Rev Microbiol 16:263–276. 10.1038/nrmicro.2018.9 29398704 10.1038/nrmicro.2018.9

[CR36] Lamprea Pineda, P.A., Demeestere K.,González-Cortés, J.J., Alvarado-Alvarado, A. A., Boon, N., Devlieghere, F., Langenhove, H. Van, & Walgraeve, C. (2023). Effect of inoculum type, packing material and operational conditions on the biofiltration of a mixture of hydrophobic volatile organic compounds in air. Sci Total Environ, 904, 167326. 10.1016/j.scitotenv.2023.167326 10.1016/j.scitotenv.2023.167326 37748600

[CR37] Lebrero R, Rodríguez E, Martin M, García-Encina PA, Muñoz R (2010) H_2_S and VOCs abatement robustness in biofilters and air diffusion bioreactors: a comparative study. Water Res 44(13):3905–3914. 10.1016/j.watres.2010.05.008 20639014 10.1016/j.watres.2010.05.008

[CR38] Lin S, Mackey HR, Hao T, Guo G, van Loosdrecht MCM, Chen G (2018) Biological sulfur oxidation in wastewater treatment: a review of emerging opportunities. Water Res 143:399–415. 10.1016/j.watres.2018.06.051 29986249 10.1016/j.watres.2018.06.051

[CR39] Liu T, Dong H, Zhu Z, Shang B, Yin F (2017) Effects of biofilter media depth and moisture content on removal of gases from a swine barn. J Air Waste Manage Assoc 67(12):1288–1297. 10.1080/10962247.2017.1321591 10.1080/10962247.2017.1321591 28453404

[CR40] López R, Cabeza IO, Giráldez I, Díaz MJ (2011) Biofiltration of composting gases using different municipal solid waste-pruning residue composts: monitoring by using an electronic nose. Bioresour Technol 102(17):7984–7993. 10.1016/j.biortech.2011.05.085 21704517 10.1016/j.biortech.2011.05.085

[CR41] Maestre JP, Gamisans X, Gabriel D, Lafuente J (2007) Fungal biofilters for toluene biofiltration: evaluation of the performance with four packing materials under different operating conditions. Chemosphere 67:684–692. 10.1016/j.chemosphere.2006.11.004 17184815 10.1016/j.chemosphere.2006.11.004

[CR42] Maia GD, Gates RS, Taraba JL (2012) Ammonia biofiltration and nitrous oxide generation during the start-up of gas-phase compost biofilters. Atmos Environ 46:659–664. 10.1016/j.atmosenv.2011.10.019

[CR43] Malhautier L, Khammar N, Bayle S, Fanlo JL (2005) Biofiltration of volatile organic compounds. Appl Microbiol Biotechnol 68:16–22. 10.1007/s00253-005-1960-z 15803311 10.1007/s00253-005-1960-z

[CR44] Márquez P, Herruzo-Ruiz AM, Siles JA, Alhama J, Michán C, Martín MA (2021) Influence of packing material on the biofiltration of butyric acid: a comparative study from a physico-chemical, olfactometric and microbiological perspective. J Environ Manag 294:113044. 10.1016/j.jenvman.2021.113044 10.1016/j.jenvman.2021.113044 34130131

[CR45] Márquez P, Siles JA, Gutiérrez MC, Alhama J, Michán C, Martín MA (2022) A comparative study between the biofiltration for air contaminated with limonene or butyric acid using a combination of olfactometric, physico-chemical and genomic approaches. Process Saf Environ Prot 160:362–375. 10.1016/j.psep.2022.02.024

[CR46] Ministerio de Vivienda, Ciudad y Territorio de Colombia (2014) Decree 1287. https://minvivienda.gov.co/normativa/decreto-1287-2014. Accessed 23 May 2024

[CR47] Muccee F, Ejaz S, Riaz N (2019) Toluene degradation via a unique metabolic route in indigenous bacterial species. Arch Microbiol 201(10):1369–1383. 10.1007/s00203-019-01705-0 31332474 10.1007/s00203-019-01705-0

[CR48] Mulvaney RL (1996) Nitrogen—inorganic forms. In: Methods of Soil Analysis Part 3—Chemical Methods. Soil Science Society of America, American Society of Agronomy, pp 1123–1184. 10.2136/sssabookser5.3.c38

[CR49] Muñoz R, Malhautier L, Fanlo JL, Quijano G (2015) Biofiltration of volatile organic compounds. Appl Microbiol Biotechnol 68:16–22. 10.1007/s00253-005-1960-z 10.1007/s00253-005-1960-z 15803311

[CR50] Occupational Safety and Health Administration (2020) OSHA occupational chemical database AMMONIA. United States Department of Labor https://www.osha.gov/chemicaldata/623 Accessed 02 Dec 2022

[CR51] Omri I, Aouidi F, Bouallagui H, Godon JJ, Hamdi M (2013) Performance study of biofilter developed to treat H_2_S from wastewater odour. Saudi J Biol Sci 20(2):169–176. 10.1016/j.sjbs.2013.01.005 23961233 10.1016/j.sjbs.2013.01.005 PMC3730789

[CR52] Ondarts M, Hort C, Sochard S, Platel V, Moynault L, Seby F (2012) Evaluation of compost and a mixture of compost and activated carbon as biofilter media for the treatment of indoor air pollution. Environ Technol 33:273–284. 10.1080/09593330.2011.570793 22519112 10.1080/09593330.2011.570793

[CR53] Park BG, Shin WS, Jeong YS, Chung JS (2008) Simultaneous removal of H_2_S, NH_3_ and Toluene in a biofilter packed with Zeocarbon carrier. J Environ Sci 17:7–17. 10.5322/jes.2008.17.1.007

[CR54] Pokorna D, Zabranska J (2015) Sulfur-oxidizing bacteria in environmental technology. Biotechnol Adv 33(6):1246–1259. 10.1016/j.biotechadv.2015.02.007 25701621 10.1016/j.biotechadv.2015.02.007

[CR55] Sharma D, Varma VS, Yadav KD, Kalamdhad AS (2017) Evolution of chemical and biological characterization during agitated pile composting of flower waste. Int J Recycl Org Waste Agric 6:89–98. 10.1007/s40093-017-0155-9

[CR56] Smalheiser NR (2017) Chapter 12 - nonparametric tests. In: Smalheiser NR (ed) Data Literacy. Academic Press, pp 157–167. 10.1016/B978-0-12-811306-6.00012-9

[CR57] Sun Y, Quan X, Chen J, Yang F, Xue D (2002) Toluene vapour degradation and microbial community in biofilter at various moisture content. Process Biochem 38:109–113. 10.1016/S0032-9592(02)00056-0

[CR58] Tian H, Liu J, Zhang Y, Yue P (2023) A novel integrated industrial-scale biological reactor for odor control in a sewage sludge composting facility: Performance, pollutant transformation, and bioaerosol emission mechanism. Waste Manag 164(11):9–19. 10.1016/j.wasman.2023.03.021 37185067 10.1016/j.wasman.2023.03.021

[CR59] Topp E, Millar S, Bork H, Welsh M (1998) Effects of marigold (Tagetes sp.) roots on soil microorganisms. Biol Fertil Soils 27:149–154. 10.1007/s003740050413

[CR60] Vela-Aparicio D, Forero DF, Hernández MA, Brandão PFB, Cabeza IO (2020) Simultaneous biofiltration of H_2_S and NH_3_ using compost mixtures from lignocellulosic waste and chicken manure as packing material. Environ Sci Pollut Res 28:24721–24730. 10.1007/s11356-020-10817-w 10.1007/s11356-020-10817-w 32951172

[CR61] Vela-Aparicio DG, Bautista CJ, Forero DF, Acevedo P, Brandão PFB, Cabeza IO (2022) Inoculation of compost biofilter for the simultaneous removal of H_2_S and NH_3_ under transient conditions of gas concentration. Chem Eng Trans 93:157–162. 10.3303/CET2293027

[CR62] Vela-Aparicio DG, Forero DF, Fernandez A, Hernandez MA, Brandao PF, Cabeza IO (2021) Operational parameters analysis for the removal of H_2_S and NH_3_ under transient conditions by a biofiltration system of compost beds. Chem Eng Trans 85:163–168. 10.3303/CET2185028

[CR63] Widiana DR, Wang YF, You SJ, Yang HH, Wang LC, Tsai JH, Chen HM (2019) Air pollution profiles and health risk assessment of ambient volatile organic compounds above a municipal wastewater treatment plant, Taiwan. Aerosol Air Qual Res 19:375–382. 10.4209/aaqr.2018.11.040

[CR64] Xie B, Liang SB, Tang Y, Mi WX, Xu Y (2009) Petrochemical wastewater odor treatment by biofiltration. Bioresour Technol 100(7):2204–2209. 10.1016/j.biortech.2008.10.035 19056260 10.1016/j.biortech.2008.10.035

[CR65] Yoon IK, Kim CN, Park CH (2002) Optimum operating conditions for the removal of volatile organic compounds in a Compost-Packed biofilter. Korean J Chem Eng 19:954–959. 10.1007/BF02707217

[CR66] Yuan J, Du L, Li S, Yang F, ZhangZ Li G, Wang G (2019) Use of mature compost as filter media and the effect of packing depth on hydrogen sulfide removal from composting exhaust gases by biofiltration. Environ Sci Pollut Res 26:3762–3770. 10.1007/s11356-018-3795-z 10.1007/s11356-018-3795-z 30539397

