# Supplementary material for: Simultaneous biofiltration of H2S, NH3, and toluene using compost made of chicken manure and sugarcane bagasse as packing material
Source: Environ Sci Pollut Res Int. 2024 Jun 26;32(48):27886–901. doi: 10.1007/s11356-024-33757-1 (PMC12695926; doi:10.1007/s11356-024-33757-1)
Supplement: Supplementary file 1 — Fig. S1 NH3 and VOCs concentrations (ppm) in relation to H2S concentration (ppm) found at El Salitre WWTP. Fig. S2 Reactor for a composting mix of sugar cane bagasse and manure. Fig. S3 Gas generation system assembly. Fig. S4 Biofilter assembly. Fig. S5 Calibration curve Na2S concentration (mg/L) added to HCl to obtain a desire concentration of H2S gas stream (ppm). Table 1 Most abundant organic compounds of the pre-treatment zone at El Salitre WWTP. Table 2 Removal efficiency (%) for H2S, NH3 and toluene in the different phases (DOCX 727 kb) [file 11356_2024_33757_MOESM1_ESM.docx]

**Simultaneous biofiltration of H_2_S, NH_3_ and toluene using compost made of chicken manure and sugarcane bagasse as packing material**

A.M. Guzmán-Beltrán^1,2^, D. G. Vela-Aparicio^2^, S. Montero^3^, I. O. Cabeza^4^, P. F. B. Brandão^2^

^1^Universidad Nacional de Colombia - Sede Bogotá - Facultad de Ingeniería - Departamento de Ingeniería Química y Ambiental- Grupo GERMINA- Bogotá D.C., Colombia

e-mail: anmguzmanbe@unal.edu.co

^2^Universidad Nacional de Colombia - Sede Bogotá-Facultad de Ciencias-Departamento de Química - Grupo GERMINA - Bogotá D.C., Colombia

e-mails: dgvelaa@unal.edu.co; pfdeb@unal.edu.co

^3^Universidad Santo Tomás - Facultad de Ingeniería Ambiental, Bogotá D.C., Colombia

e-mail: sergiomontero@usantotomas.edu.co

^4^Universidad de la Sabana  - Faculty of Engineering,Energy, Materials and Environment Laboratory, Campus Universitario Puente del Común, Km. 7 Autopista Norte de Bogotá, Chía, Cundinamarca, Colombia

  e-mail: ivan.cabeza@unisabana.edu.co

**Corresponding author:** I. O. Cabeza [ivan.cabeza@unisabana.edu.co](mailto:ivan.cabeza@unisabana.edu.co) Phone number:+57 300 3846347

**Supplementary material**

**Fig. S1** NH_3_ and VOCs concentrations (ppm) in relation to H_2_S concentration (ppm) found at El Salitre WWTP.


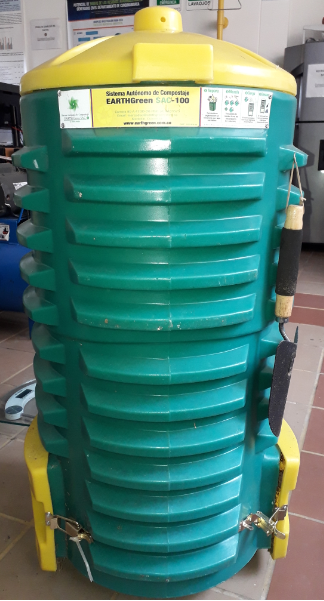


**Fig. S2** Reactor for a composting mix of sugar cane bagasse and manure.


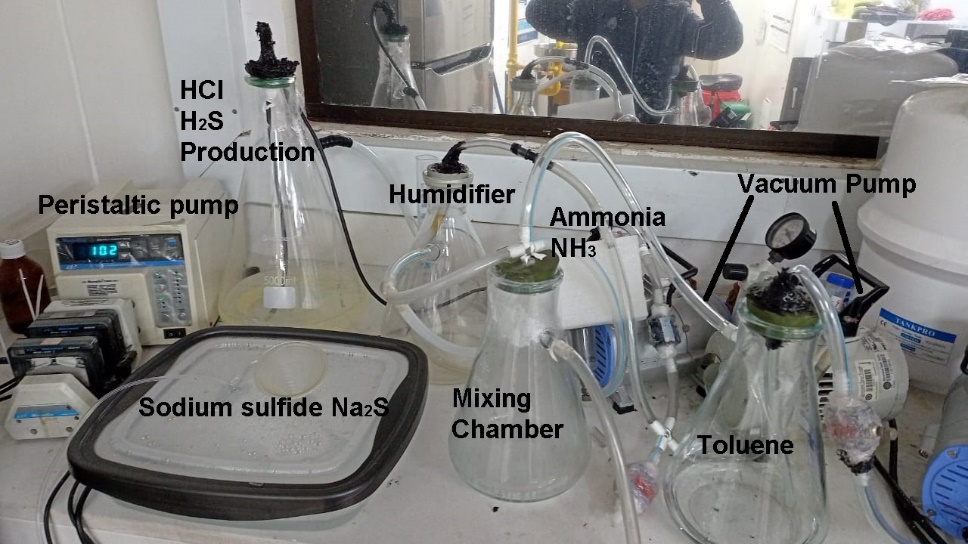


**Fig. S3** Gas generation system assembly.


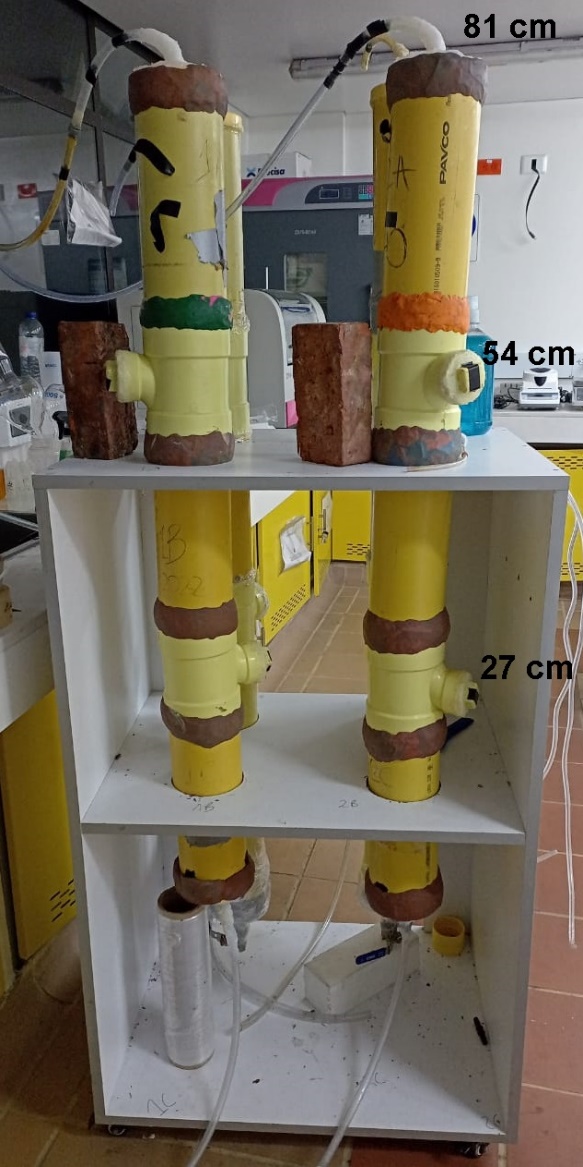


**Fig. S4** Biofilter assembly.

**Table 1** Most abundant organic compounds of the pre-treatment zone at El Salitre WWTP.

| **Retention time (min)** | **Compound** | **Molar abundancy (%)** |
| --- | --- | --- |
| 24,484 | Toluene | 3,99 |
| 31,334 | Xylene | 6,29 |
| 37,573 | Decane | 4,13 |
| 40,483 | Limonene | 2,99 |
| 37,086 | 1-ethyl-2-methylbenzene | 3,10 |
|  | 1-ethyl-3-methylbenzene |  |
| 39,238 | 1,2,3-trimethylbenzene | 3,36 |
|  | 1,2,5-trimethylbenzene |  |
| 43,280 | Undecane | 2,64 |

**Fig. S5** Calibration curve Na_2_S concentration (mg/L) added to HCl to obtain a desire concentration of H_2_S gas stream (ppm)

**Table 2** Removal efficiency (%) for H_2_S, NH_3_ and toluene in the different phases

| **Phase** | **Day** | **H_2_S RE (%)** | **NH_3_ RE (%)** | **Toluene RE (%)** |
| --- | --- | --- | --- | --- |
| I | 0-25 | 100,0 | 30 ± 5 | 33.2 ± 4.7 |
| II | 26-43 | 100 ± 1 | 68 ± 2 | 80.0 ± 2.1 |
| III | 44-60 | 78.0 ± 2.2 | 47 ± 3 | 65.6 ± 2.0 |
| IV | 60-72 | 91.9 ± 2.0 | 64 ± 3 | 73.8 ± 3.1 |
| V | 73-83 | 96.6 ±1,2 | 55 ± 4 | 71.5 ± 4.0 |
| VI | 84-89 | 71.4 ± 4.3 | 47 ± 5 | 34.5 ± 6.5 |
| VII | 90-103 | 97.0 ± 0.9 | 50 ± 5 | 52.9 ± 3.9 |
